# WATCH-BPM—Comparison of a WATCH-Type Blood Pressure Monitor with a Conventional Ambulatory Blood Pressure Monitor and Auscultatory Sphygmomanometry

**DOI:** 10.3390/s23218877

**Published:** 2023-10-31

**Authors:** Mathini Vaseekaran, Sven Kaese, Dennis Görlich, Marcus Wiemer, Alexander Samol

**Affiliations:** 1 Department of Cardiology and Critical Care Medicine, Johannes Wesling University Hospital, 32429 Minden, Germany; mathini.vaseekaran@muehlenkreiskliniken.de (M.V.); sven.kaese@muehlenkreiskliniken.de (S.K.); marcus.wiemer@muehlenkreiskliniken.de (M.W.); 2 Institute of Biostatistics and Clinical Research, University Münster, 48149 Muenster, Germany; dennis.goerlich@ukmuenster.de; 3 Department of Cardiology and Angiology, St. Antonius-Hospital Gronau GmbH, Möllenweg 22, 48599 Gronau, Germany

**Keywords:** smartwatch, hypertension, out-patient monitoring, cardiovascular diagnostics, telemedicine

## Abstract

Background: Smart devices that are able to measure blood pressure (BP) are valuable for hypertension or heart failure management using digital technology. Data regarding their diagnostic accuracy in comparison to standard noninvasive measurement in accordance to Riva-Rocci are sparse. This study compared a wearable watch-type oscillometric BP monitor (Omron HeartGuide), a wearable watch-type infrared BP monitor (Smart Wear), a conventional ambulatory BP monitor, and auscultatory sphygmomanometry. Methods: Therefore, 159 consecutive patients (84 male, 75 female, mean age 64.33 ± 16.14 years) performed observed single measurements with the smart device compared to auscultatory sphygmomanometry (n = 109) or multiple measurements during 24 h compared to a conventional ambulatory BP monitor on the upper arm (n = 50). The two BP monitoring devices were simultaneously worn on the same arm throughout the monitoring period. In a subgroup of 50 patients, single measurements were also performed with an additional infrared smart device. Results: The intraclass correlation coefficient (ICC) between the difference and the mean of the oscillometric Omron HeartGuide and the conventional method for the single measurement was calculated for both systole (0.765) and diastole (0.732). This is exactly how the ICC was calculated for the individual mean values calculated over the 24 h long-term measurement of the individual patients for both systole (0.880) and diastole (0.829). The ICC between the infrared device and the conventional method was “bad” for SBP (0.329) and DBP (0.025). Therefore, no further long-term measurements were performed with the infrared device. Conclusion: The Omron HeartGuide device provided comparable BP values to the standard devices for single and long-term measurements. The infrared smart device failed to acquire valid measurement data.

## 1. Introduction

Hypertension is one of the main risk factor for cardiovascular diseases [1,2], which are the most common cause of death in industrialized countries [3]. Multiple factors are associated with hypertension. Individuals with higher values of body mass index (BMI) are more likely to develop hypertension, suggesting the importance of adiposity in the development of hypertension [4]. The risk of hypertension, especially isolated systolic hypertension, increases at an older age. Isolated systolic hypertension is the predominant type of hypertension in the elderly and is associated with cardiovascular complications such as stroke, coronary heart disease, and heart failure. Arterial stiffness, endothelial function, atherosclerosis, and oxidative stress increase with age [5]. Target blood pressure (BP) goals need to be individualized, according to comorbidities, hypertension-mediated organ damage, coexistence of cardiovascular risk factors (including age), frailty in older patients, and individual tolerability [6]. People with hypertension often do not notice their increased BP, because of its lesser or asymptomatic character or as they measure their BP rarely, even if they know about the presence of hypertension [7,8]. One possible reason for this might be a lack of adequate BP monitoring equipment at home. Another reason could be that BP measurement with the BP measuring devices currently available on the market is too time-consuming for patients. For example, the cuff has to be put on, connected to the monitor, and the BP values have to be stored manually or written down. Meanwhile, the possibilities of using smart technologies in the health sector are constantly increasing [9,10,11]. The integration of BP measurement technology in a smart device enables flexible and uncomplicated BP measurement in most conceivable situations in daily practice [12]. The use of a special watch thus might enable adequate BP surveillance and correspondingly targeted therapy initiation or optimization. In addition, this device could also simplify the monitoring and optimization of heart failure therapy in an out-patient setting, through patient-controlled self-measurements [10]. The use of smart devices for BP monitoring and therapy is not yet standard and poorly researched. Therefore, this study investigated two different smart devices.

The Omron HeartGuide device is a new smart device with an integrated oscillometric BP measurement tool in its wristband, and it is able to store recorded measurements and enables connection to personal smartphones [13,14]. The Smart Wear watch, on the other hand, measures BP using infrared. The aim of this study was to investigate the reproducibility of the BP values recorded by conventional BP monitors using the Omron HeartGuide and a Smart Wear watch for single measurements and for long-term use distributed throughout the day.

In this study, we hypothesized that the BP values recorded by smart devices would correlate with the BP values recorded by conventional BP monitors, which in turn means that smart devices would be an alternative to the conventional blood pressure measurement methods.

## 2. Methods

**Figure 1:**
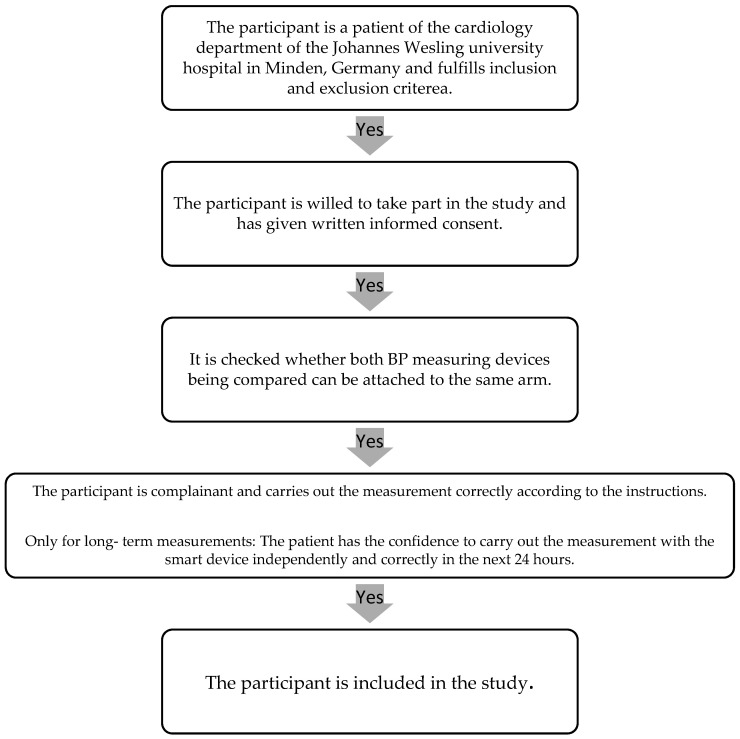
Participant selection process.

The BP of 109 consecutive patients on the cardiology ward of the Johannes Wesling university hospital in Minden, Germany, was measured in accordance with Riva-Rocci and compared with a single BP measurement using the smart device (Figure 1). A subgroup of 50 patients had a third measurement with an infrared smart device.

In addition, 50 consecutive patients who received 24 h BP monitoring for diagnostic purposes were equipped with an Omron HeartGuide watch over this period. These patients were asked to use the smart device to measure their BP one minute after the BP monitor had measured the BP on the upper arm. The recordings of the BP monitor were read out after 24 h. The measured values were then compared with the data from the long-term BP measurement. Due to the invalid data of the single measurement with the infrared device, we performed no further long-term measurements comparing this device with standard BP monitor. All participants provided their written informed consent to participate. The study was approved by the local ethics committee (AZ:2020-647).

### 2.1. Study Population for the Single Measurements and Long-Term Measurement

All participants for the single measurement were patients in the cardiology department of the Johannes Wesling university hospital. Adult outpatients who were scheduled to conduct a 24 h long-term BP measurement in clinical practice were consecutively recruited at the cardiology department of the Johannes Wesling university hospital in Minden, Germany, for the long-term measurement. The inclusion criterion was age ≥18 years. The exclusion criteria were pregnancy and breast-feeding period. These criteria were selected in accordance to the recommendations of our local ethics committee.

### 2.2. Participant Selection Process

The participant selection process was performed using the following flow diagramm (Figure 1):

### 2.3. BP Measurement Devices

The Omron HeartGuide device (Omron Healthcare Co., Ltd., Kyoto, Japan) is a patient-self-initiated automatic oscillometric device for measuring BP at the wrist [14]. In this study we used the Omron HeartGuide model HEM-6411T-MAE. We chose this device because it was the most recent model capable of measuring blood pressure oscillometrically at the time of the start of the study. No prior investigations were carried out. The wrist should be at heart level when BP is measured, to reduce hydrostatic effects [14]. The cuff of this device is inflated automatically by an electric pump and then deflated by a mechanical valve. The device analyzes the pulse wave detected during inflation using an algorithm for determining the BP. During the inflation period of the cuff, it measures the systolic BP (range of 60–230 mmHg), the diastolic BP (range of 40–160 mmHg), and the pulse rate (range of 40–180 beats per min) [15].

We used the HeartGuide device cuff size that is appropriate for those with wrist circumferences in the range of 16.0–19.0 cm. The BP values measured by the watch were transferred via Bluetooth to a smartphone on which the Omron HeartAdvisor app was installed. All BP values are listed with the date and time on the smartphone or in the app. These lists can be sent to a PC via the app [16]. All data were stored pseudonymized on the PC in a file (format MS excel).

The TV962SM smart device (Smart Wear, Fuzhou, China) is also worn on the wrist. We chose this device because it was a cheap alternative to the Omron device, with an online reputation of valid BP measurement. Again, no prior studies were conducted. This device measures and analyzes with infrared light the pulse wave that emanates from the heart. Infrared light is absorbed by the blood in the arteries more than by surrounding tissue or bone, and this allows BP to be measured by changing the intensity of the light. A calculation based on signal-processing algorithms is superimposed on the sensor-based measurement, which ultimately makes it possible to estimate the BP. The reference point is the pulse [17,18]. The pulse transit time (PTT) is determined. Pulse transit time is the time from the onset of a pulse wave due to heart contraction to the point of arriving in a peripheral part of the body. The beginning of a pulse wave is determined by the time of heart contraction using the R-wave from electrocardiogram (ECG) recordings to define the initiation. In addition, the highest and lowest values of the photoplethysmography signal (PPG) are measured at the wrist with the help of sensors in the smart device—in other words, the change in vessel width that also accompanies the pulse wave at small vessels. The higher the BP, the more tense the vessels and the faster the pulse wave.

The 24 h long-term BP measurements were recorded using an automatic oscillometric device with a BP cuff worn on the upper arm (ambulatory BP monitor). After the 24 h had elapsed, the data were read out by qualified medical staff. The results were visualized in table format.

The single BP measurements were performed using a conventional BP cuff and a stethoscope.

### 2.4. BP Measurement Protocol in the Single Measurements

**Figure 2:**
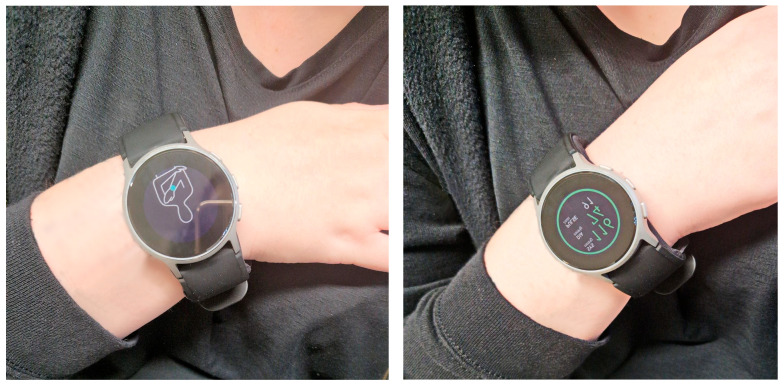
Measurement procedure: The device is positioned at heart level in accordance to the instructions on the display (**left**); after the measurement, blood pressure values are displayed (**right**).

The participants were seated in a quiet room at a comfortable room temperature, with their back supported, their legs uncrossed, and the measurement arm supported so that the wrist was at heart level. The BP measurements were started after a resting period of five minutes. Then the wrist circumference was measured. For the BP measurements using a standard manual sphygmomanometer, qualified medical stuff measured the participant’s BP according to Riva-Rocci using a stethoscope and a calibrated sphygmomanometer. Then, 60 s after the measurements using the standard sphygmomanometer, the BP was measured using the Omron HeartGuide. The measurement with the Omron HeartGuide was instructed and observed by a qualified medical staff member. A visualization of the BP measurement procedure is shown in Figure 2.

In the 50 patients of the single measurement, a third measurement was performed using the infrared Smart Wear watch. Therefore, the participants remained in a sitting position. After the measurement with the Omron HeartGuide, the watch was removed and the smart-wear watch was put on the same wrist. After one minute of resting time the BP was measured. Therefore, the patients were asked not to move and keep their arm in a comfortable relaxed position on their lap. The end of measurement was indicated by a vibration of the device, and the value was displayed. After the inclusion of 50 patients, an interim analysis was performed.

### 2.5. BP Measurement Protocol in the Long-Term Measurements

The Omron HeartGuide (at the wrist) and the ambulatory BP monitor (at the upper arm) were worn on the same arm. The ambulatory BP monitor measured the BP automatically every 30 min at daytime (6.00 a.m. to 10.00 p.m.) and every 60 min at night time (10.00 p.m. to 6.00 a.m.). The patients were instructed how to measure the BP with the Omron HeartGuide and asked to measure the BP one minute after the device on the upper arm had measured the BP. They were also informed which position they should take to measure in the right way. The first measurement was observed by a qualified medical stuff. All other measurements were performed without observation.

### 2.6. Statistical Analysis of the Single and Long-Term Measurements

The statistical analyses of the single measurement and long-term measurement data were broadly similar and described only once here. The primary outcome for both were systolic blood pressure values (SBP) and diastolic blood pressure values (DBP). From the long-term measurements (24 h), we computed the average over 24 h, as an aggregated measure for the primary outcome. The SBP and DBP of all measurement methods were described using means and standard deviation. Furthermore, the two different devices (Omron HeartGuide, Smart Wear) were compared to the Riva-Rocci (standard clinical assessment of BP) as the mean and standard deviation of the patient specific differences. Associations of baseline variables (age, BMI, and wrist circumference) with SBP and DBP were analyzed using Spearman rho correlation. The reliability of the smart watches was assessed using Bland–Altman plots against the Riva-Rocci measurement. For each patient, the difference between the respective smart watch and the RR measurement was plotted against the mean of the two values. In this way, systematic bias in terms of general under or overestimation of BP was assessed. Bland–Altman plots were presented with the mean difference as solid horizontal line and the lower and upper 1.96 standard deviations of the mean difference. The Bland–Altman plots were then used to assess whether all/most measured values were within the tolerance ranges, i.e., the lower and upper 1.96 standard deviations of the mean difference. If this was the case, the measuring method with the smart device was attested to have sufficient accuracy compared to the standard measuring method. As additional information on the association between the BP measurement methods, the two-way mixed intra class correlation coefficient was calculated. The ICC were interpreted according to Cicchetti, and Koo and Li:

According to Cicchetti, ICC values less than 0.4 are indicative of poor reliability (“bad”), values between 0.4 and 0.59 indicate moderate reliability (“average”), values between 0.6 and 0.74 indicate good reliability (“good”), and values greater than 0.74 indicate excellent reliability (“very good”). According to Koo and Li, ICC values less than 0.5 are indicative of poor reliability (“bad”), values between 0.5 and 0.75 indicate moderate reliability (“average”), values between 0.75 and 0.9 indicate good reliability (“good”), and values greater than 0.90 indicate excellent reliability (“very good”).

Additionally, for the long-term measurement, the non-aggregated data were compared between the smart watch device and the clinical long-term measurements using computing differences. The differences were displayed as a scatter plot over time (24 h) and the general trend was assessed using LOESS regression. No inference statistics were computed for this exploratory analysis.

## 3. Results

### 3.1. Single Measurement

Patient characteristics are listed in Table 1. A total of 109 participants were screened for the validation studies. We excluded 9 participants for not obtaining any blood pressure readings, resulting in a final participant group of 53 men (53%) and 47 women (47%) undergoing the study. Their average age was 69.8 ± 13.6 years (mean ± SD). The mean wrist circumference was 17.9 ± 1.6 cm. The mean BMI was 27.8 ± 5.5 kg/m^2^.

**Figure 3:**
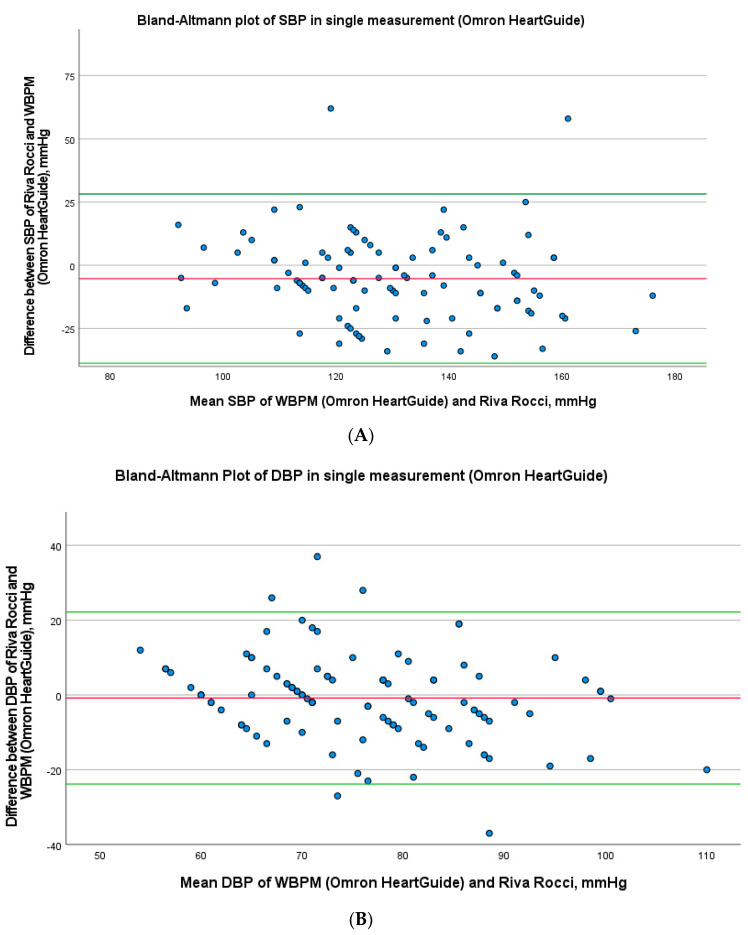

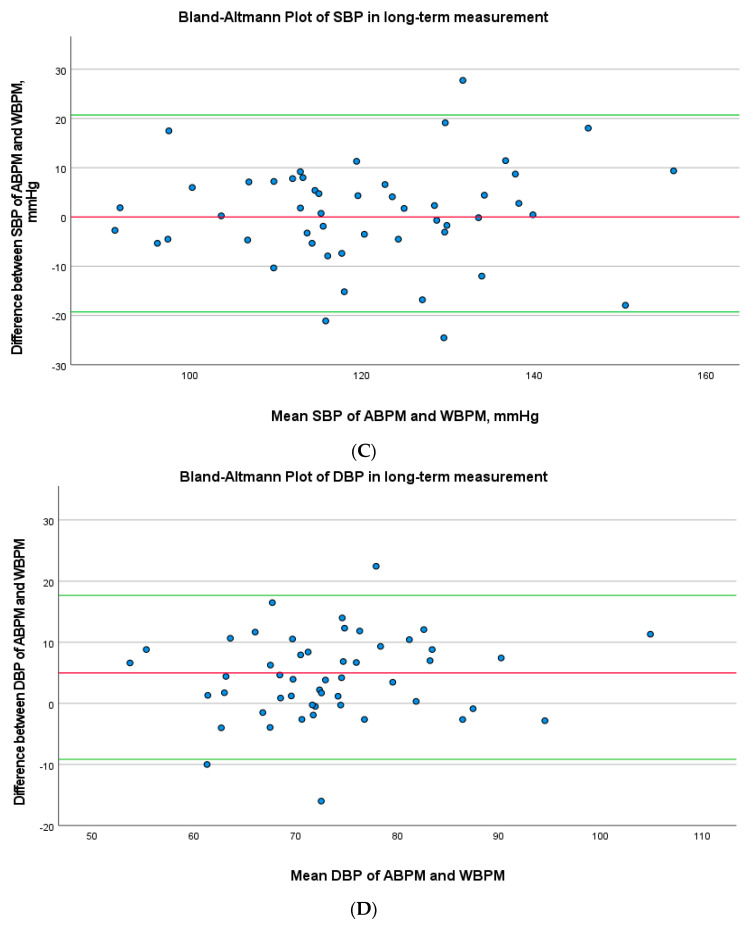

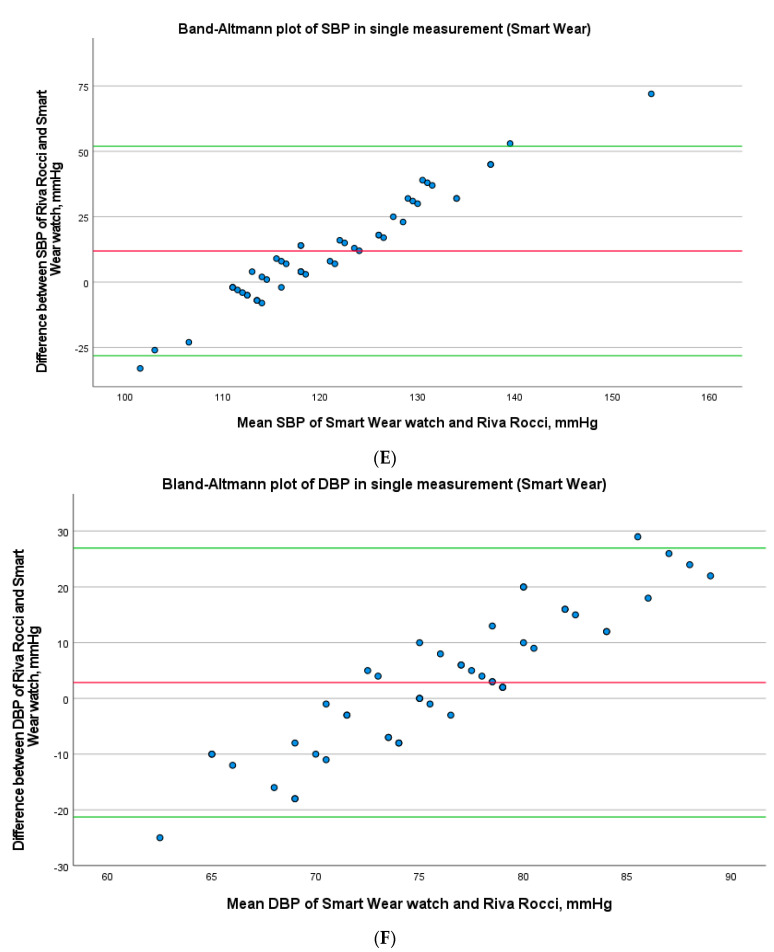
(**A**) Bland–Altman Plot of SBP in single measurement (Omron HeartGuide). Bland–Altman plots for the SBP differences between Riva-Rocci and readings of the WBPM in the single measurement. Thick red solid line = mean difference; green line = ±1.96 standard deviations of the mean difference. Abbreviations: WBPM, wearable blood pressure monitoring; SBP, systolic blood pressure. (**B**) Bland–Altman plot of DBP in single measurement (Omron HeartGuide). Bland–Altman plots for the DBP differences between Riva-Rocci and readings of the WBPM in the single measurement. Thick red solid line = mean difference; green line = ±1.96 standard deviations of the mean difference. Abbreviations: WBPM, wearable blood pressure monitoring; DBP, diastolic blood pressure. (**C**) Bland–Altman Plot of SBP in long-term measurement. Bland–Altman plots for the SBP differences between ABPM and readings of the WBPM in the long-term measurement. Thick red solid line = mean difference; green line = ±1.96 standard deviations of the mean difference. Abbreviations: WBPM, wearable blood pressure monitoring; ABPM, ambulatory blood pressure monitoring; SBP, systolic blood pressure. (**D**) Bland–Altman plot of DBP in long-term measurement. Bland–Altman plots for the DBP differences between ABPM and readings of the WBPM in the long-term measurement. Thick red solid line = mean difference; green line = ±1.96 standard deviations of the mean difference. Abbreviations: WBPM, wearable blood pressure monitoring; ABPM, ambulatory blood pressure monitoring; DBP, diastolic blood pressure. (**E**) Bland–Altman Plot of SBP in single measurement (Smart Wear watch). Bland–Altman plots for the SBP differences between Riva-Rocci and readings of the Smart Wear watch in the single measurement. Thick red solid line = mean difference; green line = ±1.96 standard deviations of the mean difference. Abbreviations: SBP, systolic blood pressure. (**F**) Bland–Altman Plot of DBP in single measurement (Smart Wear watch). Bland–Altman plots for the DBP differences between Riva-Rocci and readings of the Smart Wear watch in the single measurement. Thick red solid line = mean difference; green line = ±1.96 standard deviations of the mean difference. Abbreviations: DBP, diastolic blood pressure.

The mean difference between the systole of Omron HeartGuide and the auscultatory sphygmomanometry was −5.28 ± 1.71 mmHg and those of the diastole was −0.81 ± 1.17 mmHg (Table 2). In the Bland–Altman plot, there is an acceptable deviation in the measured values of both devices (Figure 3A,B).

The intraclass correlation coefficient (ICC) according to Cicchetti was “very good” for systolic blood pressure (SBP) and “good” for diastolic blood pressure (DBP). The ICC according to Koo and Li was “good” for SBP (0.765) and “average” for DBP (0.732). To investigate whether the differences in BP values between the individual measurement methods depended on age, BMI, or wrist circumference, Spearman rho correlation was performed. There was no correlation between the difference in the SBP or DBP of both devices and age/BMI/wrist circumference in the Spearman rho correlation. Thus, the differences in BP values did not depend on the factors involved.

In the infrared watch group, the ICC according to Cicchetti was “bad” for SBP (0.329) and for DBP (0.025). The ICC according to Koo and Li was also “bad” for the SBP and for DBP. The mean difference between the systole of the Smart Wear watch and the auscultatory sphygmomanometry was 11.92 ± 2.89 mmHg and those of the diastole was 2.86 ± 1.74 mmHg. The Bland–Altman plot is shown in Figure 3E,F. It was also noticeable that the smart device did not display any systolic values over 120 mmHg. Due to these bad results in the interims analysis of 50 patients, no further investigations were performed with this watch.

### 3.2. Long-Term Measurement

Patient characteristics are listed in Table 1. For long-term measurement, we screened consecutive patients from our outpatient clinic. In this study arm, 50 participants were screened for the validation studies. The participants belonged to Western ethnic groups. No participants were excluded, resulting in a final participant group of 27 men (54%) and 23 women (46%). Their average age was 52.3 ± 14.5 years (mean ± SD). The mean wrist circumference was 17.6 ± 1.3 cm. The mean BMI was 29.3 ± 6.1 kg/m^2^.

**Figure 4:**
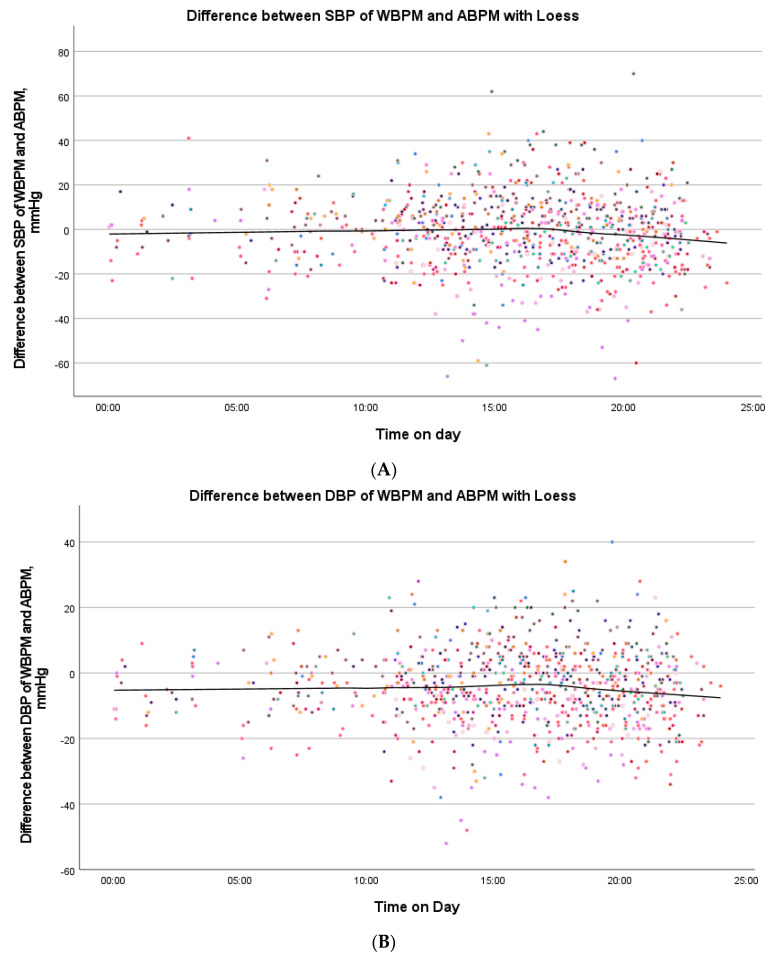
(**A**) Difference between the SBP of WBPM and ABPM with Loess. Loess plot with the SBP difference of WBPM and ABPM throughout the day in long-term measurement. Colored dots represent values of individual participants. Abbreviations: WBPM, wearable blood pressure monitoring; ABPM, ambulatory blood pressure monitoring; SBP, systolic blood pressure; DBP, diastolic blood pressure. (**B**) Difference between the DBP of WBPM and ABPM with Loess. Loess plot with the DBP difference of WBPM and ABPM throughout the day in long-term measurement. Abbreviations are similar to (**A**).

The subjects took a total of 968 measurements with the smart device. A total of 811 paired measurements of ABPM followed by WBPM were successfully obtained from the 50 participants. The mean difference between the systole of Omron HeartGuide and the ABPM was 0.72 ± 1.44 mmHg, and that of the diastole was 4.27 ± 0.97 mmHg (Table 3) The ICC according to Cicchetti was “very good” for both SBP (0.880) and DBP (0.829). The ICC according to Koo and Li was “good” for both SBP and DBP. There was no correlation between the difference of the SBP or DBP of both devices and age/BMI/wrist circumference in the Spearman rho correlation. In the Bland–Altman plot, there was an acceptable deviation in the measured values of both devices (Figure 3C,D). There was hardly any difference of SBP and DBP for both devices at different measurement times in a scatterplot with the Loess-function (Figure 4A,B). Table 4 shows recruitment details compared to the single measurement group.

## 4. Discussion

Gold standard for professional BP measurement performed by medical staff is auscultatory sphygmomanometry [19]. Different types of patient-directed measurement tools have been developed during the last decades [12]. The conventional region for cuff placement is the upper arm, and devices for sphygmomanometric BP measurement at the wrist have shown comparable results [19]. Thus, insights from patients’ BP behavior in daily life became observable for physicians and health care professionals. Nevertheless, these measurement tools are mostly deposited at home and often not available in many situations when BP disturbances or symptoms occur [20]. Additionally, the data transfer from measurement of BP by the patient and interpretation by the physicians depends on correct documentation by the patients and is in general performed with a greater or lesser relevant time delay during the next patient visit [21]. Therefore, smart technology could be a useful tool to avoid transcription errors and time delays. Validated data regarding the use of BP smart devices in daily practice are sparse. Especially, there is a lack of investigation of the usefulness of the Omron HeartGuide in Western patient populations. The biggest advantage of the Omron HeartGuide is the integration of a reliable RR measurement ability in a wearable smart device. This allows the recording of BP values in an ambulatory setting and mirrors the real life RR behavior of our patients in daily life. Thus, we are able to adjust medical therapy to true conditions at home and to react fast to changes in BP values shortly after measurement. A Japanese study had already compared WBPM using an oscillometric device (HeartGuide) and traditional ABPM. The results indicated that the mean difference between the WBPM and ABPM was acceptable in both office and out-of-office settings [14]. To our knowledge, we performed the first study using the Omron HeartGuide device in a Western population and the first comparison with an infrared smart device for BP measurement.

### 4.1. Single Measurement

The individual measurements were carried out under strict control, to obtain correct and valid measurements. However, only a snapshot of the BP was taken during this examination. We carried out and documented exactly one successful BP measurement both with the smart device and with the auscultatory method, so that, in the end, we had one pair of BP values for both devices for each study participant.

The mean difference between the systole of Omron HeartGuide and the auscultatory sphygmomanometry was −5.28 ± 1.71 mmHg and that of the diastole was −0.81 ± 1.17 mmHg. Thus, the Omron HeartGuide measured lower BP values of SBP as well as DBP than the conventional auscultatory method. This difference could be explained by the amplification of pulse pressure between brachial and radial arteries [22]. In one study, the usefulness of 27 different, exclusively oscillometric, automated devices (on the upper arm, wrist and index finger) was tested and compared to automated self-measurement and a standard method with a double tube stethoscope on the same arm. On average, the devices measured the systolic pressure between 13.2 mmHg lower and 3.5 mmHg higher than the standard procedure; the diastolic pressure was measured between 17.4 and 0.2 mmHg lower [19].

We compared our results to those from the Japanese study. The ICC for SBP was comparable between the Japanese (0.726) [14] and our study (0.765). The ICC for DBP in our analysis was slightly higher (0.732) than in the Japanese study (0.627) [14]. Moreover, the mean difference between the device on the upper arm and the device on the wrist was different in both studies. The SBP and the DBP mean difference between both devices in our study compared to the Japanese trial showed small differences but very similar results. A potential reason for these small differences could have been the use of an auscultatory method in our study, which we compared to the smart device, while the Japanese study used an automatic BP device [14]. There were some previous studies that demonstrated that BP differs by 7 mmHg if the height difference between the heart level and cuff position is 10 cm, due to hydrostatic pressure [23,24]. The Japanese study recommended, when positioning the arm or hand at heart level, ensuring that the arm is brought to heart level from above until the device vibrates and thus indicates the correct position [14]. We followed this recommendation to avoid patients lowering their arms involuntarily during the measurement, which may have resulted in false values. To avoid this, the participants were instructed to raise their arm to the upper limit of the range, relative to the heart, in which the device vibrates before initiating an ambulatory reading [14]. In the Japanese study, participants were asked to shift the WBPM device from the lower position to the upper position to set the position of the WBPM device [14]. The results of both studies described above could confirm this assumption, because diastolic values, which take longer (arm deviation from measuring range) to record compared to systolic values, were better after this improvement had been carried out.

There was also another Japanese study that compared other WBPMs (Omron HEM-6410T-ZM or Omron HEM-6410T-ZL) with a standard mercury sphygmomanometer [15]. The mean differences between the reference BPs and HEM-6410T-ZM readings were −0.9 ± 7.6 mm Hg and −1.1 ± 6.1 mm Hg for SBP and DBP according to criterion 1 of the ANSI/AAMI/ISO 81060-2:2013 guidelines [15].

The mean differences between the reference BPs and HEM-6410T-ZL readings were 2.4 ± 7.3 mm Hg and 0.7 ± 7.0 mm Hg for SBP and DBP according to criterion 1 of the same guidelines [15]. In that study, the two observers were blinded to each other’s readings, and a third observer served as a supervisor, who checked the BP readings by the two observers. The study population were healthy volunteers in that study, while we used patients in hospital, most of whom already had arterial hypertension.

### 4.2. Long-Term Measurement

The long-term study enabled the integration and examination of the device in daily life. There is growing evidence that daytime BP variability is an independent predictor of hypertensive target organ damage and cardiovascular events [25] and that work stress may increase ambulatory BP levels throughout the day [26].

We compared our results to those of the Japanese study, which was the first study to compare WBPM using an oscillometer device (HeartGuide) and traditional ABPM. The ICC for SBP was higher in our study (0.880) than in the Japanese study (0.640) [14]. The ICC for DBP was higher (0.829) than in the Japanese study (0.646) [14]. In the Japanese study there was a SBP mean difference between both devices of 3.2 ± 17.0 and a DBP difference of −3.2 ± 11.3 (11). In our study the SBP mean difference was lower (0.72 ± 1.44), but the DBP was higher (4.27 ± 0.97) (Table 3). As described in the discussion of the single measurement, the arm position or movement suggested in the Japanese study was also adopted in the long-term study. Participants were asked to measure the BP as often as possible, even at nighttime. Furthermore, the participants were asked to stick to a time difference of 60 s between the two measurements with the two devices, to avoid systemic bias of the values due to the automatic measurements. The delay between the automatic and the smart device measurement in the Japanese study was not clarified, this may explain why our results were different.

### 4.3. Smart Wear Watch

Unlike the Omron HeartGuide, which measures BP with the help of a cuff system, the Smart Wear watch measures and analyzes the pulse wave that emanates from the heart with infrared light. As reported previously [22,27,28,29], in order to determine the BP from cardiovascular parameters without a squeezing cuff, these values (blood flow, reflection factor or blood flow, pulse wave velocity, radius, blood density, blood viscosity, peripheral terminating resistance) must be measured or known [22]. None of these parameters were known in our study. There are also only a few studies on using this type of smart device to derive a BP [30].

### 4.4. Advantages and Disadvantages of BP Detection using the Smart Device and Outlook

A big advantage of the smart device is that it can measure BP without much effort or large BP cuffs. This makes BP measurement using this device possible everywhere.

Since the measurements with the Omron HeartGuide can only be triggered manually and the wearer of this watch has to sit down and position the arm correctly, measurements are difficult to take at night or during the sleeping period, mainly because the measurements at night in the long-term study were carried out in a more or less awake and active state. It would be a benefit in the future if this device could automatically measure BP at night without changing the position of the arm.

However, the patients who wear this smart device for diagnostic purposes must be very compliant and perform the measurement correctly. Otherwise, it falsifies the results. Easier handling of the Omron HeartGuide would be desirable in the future. Unfortunately, no automatic night measurements are currently possible with the Omron HeartGuide. Because nighttime measurements are indispensable when diagnosing hypertension or when differentiating between primary and secondary arterial hypertension by differentiating between “dippers” and “non-dippers”, this possibility would improve both the screening for arterial hypertension and the control of drug-treated hypertension [31].

The transmission process of the measured BP values through the app [16] to the smartphone was quick and safe and guaranteed a valid collection and storage process.

At present, the BP values recorded by the Omron HeartGuide can only be sent via Bluetooth to the individual smartphone of the user. For data transfer to the doctor a manually encryption and an active transfer by e-mail, for example, as a written document is necessary. This small step requires a certain amount of effort. However, if the BP values were made available directly to the doctor, virtual therapy control would be possible without personal contact.

Another disadvantage of the smart device may be that some patients could get carried away with their hypertension, by taking too many ambulatory measurements [32,33].

### 4.5. Mismeasurements

In the long-term measurement, the participants could not be checked during the period of the long-term study as to whether the measurements were carried out correctly. False measurement leads to false results. For example, participants mostly waited more than a minute before taking the measurement with the Omron HeartGuide after the measurement of the device on the upper arm.

Since the participants for the individual measurements were recruited in the department of cardiology, some patients had cardiac insufficiency with dyspnea at rest. In nine failed measurements, four participants had such severe resting dyspnea that a movement error was reported due to the strong thorax rise and fall and a measurement was not possible. Therefore, subsequent studies must determine whether the device is suitable for monitoring heart failure therapy in all stages, since heart failure is usually associated with dyspnea with heavy breathing effort.

One wheelchair user was also included in our study. He described that having the device on the upper arm, in particular, was very obstructive for the movement of the arms to push the wheelchair wheels. A device on his wrist would not bother him at all with his mobility.

### 4.6. Limitations

Our study included only a small patient collective. We recruited 50 patients for the long-term measurement and 109 patients for the single measurement. A larger patient cohort would be advantageous for a better conclusiveness. Furthermore, this study was not randomized or blinded. The measurement was carried out only by one person and not by several people, so that a comparison or statement regarding the measurement quality cannot be made. In the long-term study over 24 h, an average of only 16 BP values that could later be evaluated were recorded with the smart device. This may have been due to patient compliance.

## 5. Conclusions

The Omron HeartGuide is a suitable smart device for monitoring or controlling BP with highly reproducible BP values compared to the standard methods in clinical use. The Omron HeartGuide may allow telemetric treatment observation and improvement in an outpatient setting. As a glimpse into the future, the Omron HeartGuide could be a good alternative to a long-term BP monitor on the upper arm, according to further studies, and could therefore be used for 24 h long-term measurements, both in hospitals and in the outpatient area. The infrared smart device failed to acquire valid measurement data.

## Figures and Tables

**Table 1:** Characteristics of the study participants.

Single Measurement		Long-Term Measurement
Age, y	69.8 ± 13.6	52.3 ± 14.5
Men:women, n	57:52	27:23
Wrist circumference, cm	17.9 ± 1.6	17.6 ± 1.3
Height, meter	1.71 ± 0.1	1.75 ± 0.1
Weight, kg	81.8 ± 18.5	90.0 ± 20.9
BMI kg/m^2^	27.8 ± 5.5	29.3 ± 6.1

Data are expressed as the mean ± standard deviation or percentages or number.

**Table 2:** Comparison of BP measured using WBPM and Riva-Rocci for single measurement.

	Omron HeartGuide (WBPM)	Riva Rocci	Difference (Riva Rocci-WBPM)	Correlation Coefficient (ICC)
SBP, mmHg	132.96 ± 21.10	127.68 ± 18.89	−5.28 ± 1.71	0.765
DBP, mmHg	76.41 ± 14.11	75.60 ± 11.22	−0.81 ± 1.17	0.732

Abbreviations: BP, blood pressure: WBPM, wearable blood pressure monitoring; SBP, systolic blood pressure; DBP, diastolic blood pressure.

**Table 3:** Comparison of BP measured using WBPM and ABPM for long-term measurement.

	Omron HeartGuide (WBPM)	Ambulatory Blood Presaure Monitor	Difference (ABPM-WBPM)	Correlation Coefficient (ICC)
SBP, mmHg	119.89 ± 15.21	120.61 ± 15.75	0.72 ± 1.44	0.880
DBP, mmHg	71.28 ± 9.65	75.54 ± 10.43	4.27 ± 0.97	0.829

Abbreviations: BP, blood pressure; WBPM, wearable blood pressure monitoring; ABPM, ambulatory blood pressure monitoring; SBP, systolic blood pressure; DBP, diastolic blood pressure.

**Table 4:** Recruitment details.

	Single Measurement n = 109	Long-Term Measurement n = 50
Smoker (N=)	16	10
Cardiovascular disease	107	38
- Hypertension	93	35
- Heart failure	22	0
Antihypertensive medication	90	31

## Data Availability

Data are available from corresponding author. All authors take responsibility for all aspects of the reliability and freedom from bias of the data presented and their discussed interpretation.
